# Regression is good

**DOI:** 10.3399/bjgp16X685489

**Published:** 2016-06

**Authors:** Saul Miller

**Affiliations:** Wooler, Northumberland.

**Figure 1:**
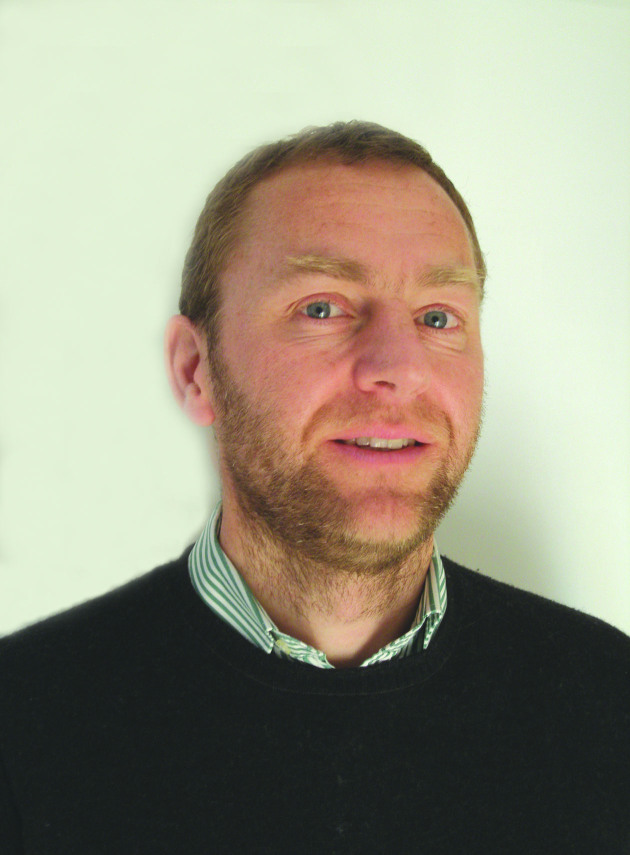


So, the headlines suggest the NHS has a whole new plan for primary care.^1^ Backed up with funding too. The outcome, apparently, will be that our share of the NHS budget will increase steadily over the next 5 years. Really? Perhaps I am just too sceptical to feel warmed by this promise of generosity. I still have hope though; it’s just that it does not stem from any announcements. Weirdly, it comes from a statistical concept.

Statistics is not generally my thing. Analyses such as the recent study comparing data from a number of national registries between 1990 and 2010 have their place of course.^2^ Many Western European countries, it transpires, have seen significant falls in this period in absolute inequalities in mortality even though relative differences between the best off and the poorest increased. The problem is, this stuff confuses me. It takes a lot of effort to deduce that mortality rates for everyone dropped, and more so for the lowest socioeconomic groups, so the life expectancy gap between the best off and the poorest really did get smaller.

As hopeful as a study like this might be then, I am not really wired to be warmed by statistical analyses the way I should. It is much easier to relate to a good story than to data.

A recent piece about self-harm is a good illustration of this point.^3^ The anonymous author has clearly self-harmed many times over a long period. She (or he) describes vividly the varied responses of medical staff to her self-inflicted hurts, and to herself more generally, when she has sought treatment. Reading her story made me distinctly uncomfortable: might I lack empathy too in such encounters? Numbers have never affected me like that.

Another example: there has been a 20% fall in the incidence of dementia compared with expectations based on data from 20 years ago, and the drop seems to be due to a better than expected outcome for men over 65.^4^ This implies my prospects of keeping my marbles in my dotage have thereby improved, provided I stay off the anticholinergics,^5^ but somehow this still leaves me unmoved. The problem is with relating to the particular — me — from the general.

Regression towards the mean, however: here is a statistical concept that really does give me a warm feeling. It is the idea that if a variable is extreme on its first measurement, it will tend to be closer to the average on its second measurement; and it holds for the opposite case too.

Primary care had a high point in its share of the NHS budget in 2004–2005 following the introduction of the Quality and Outcomes Framework (QOF), but has slid since to its lowest ebb.^6^ Regression suggests it is likely to improve in future.

Trusting to this gives me far more hope than any headline.

## References

[1] NHS England NHS England backs general practice with a multi-billion transformation plan. https://www.england.nhs.uk/2016/04/gpfv/.

[2] Mackenbach JP, Kulhánová I, Artnik B (2016). Changes in mortality inequalities over two decades: register based study of European countries. BMJ.

[3] Self harm and the emergency department (2016). BMJ.

[4] Matthews FE, Stephan BC, Robinson L, Cognitive Function and Ageing Studies (CFAS) Collaboration (2016). A two decade dementia incidence comparison from the Cognitive Function and Ageing Studies I and II. Nat Commun.

[5] Risacher SL, McDonald BC, Tallman EF (2016). Association between anticholinergic medication use and cognition, brain metabolism, and brain atrophy in cognitively normal older adults. JAMA Neurol.

[6] Soteriou M (2013). RCGP demands 11% share of NHS funding for GPs. GP.

